# Intraperitoneal administration of NMDA-Subunit NR1-receptor antibodies does not improve long-term outcome in a murine MCAo-stroke model

**DOI:** 10.3389/fnins.2025.1614924

**Published:** 2025-07-07

**Authors:** Carolin Albrecht, Christoph Harms, Jakob Kreye, Matthias Endres, Susanne Mueller, Philipp Boehm-Sturm, Stefan Paul Koch, Dorette Freyer, Harald Prüss, Samuel Knauss

**Affiliations:** ^1^Charité Universitätsmedizin Berlin, Corporate Member of Freie Universität Berlin and Humboldt-Universität zu Berlin, and Berlin Institute of Health; Charité 3R – Replace | Reduce | Refine, Berlin, Germany; ^2^German Centre for Cardiovascular Research (DZHK), Partner Site Berlin, Berlin, Germany; ^3^Center for Stroke Research Berlin, Charité Universitätsmedizin Berlin, Berlin, Germany; ^4^German Center for Neurodegenerative Diseases (DZNE), partner site Berlin, Berlin, Germany; ^5^German Center for Mental Health (DZPG), Partner site Berlin, Berlin, Germany; ^6^Charité-Universitätsmedizin Berlin, Charité Core Facility Experimental MRIs, Berlin, Germany

**Keywords:** antibodies, excitotoxicity, MCAo, NMDA-receptor, stroke

## Abstract

Ischemic stroke is a major cause of disability worldwide, and current treatment is largely limited to thrombolytics. Therefore, additional therapeutic strategies are warranted. Previous evidence suggests that NMDA receptor antibodies targeting specific subunits may reduce excitotoxicity and lesion size. This study evaluates the effects of a specific NMDAR-NR1 antibody in both *in vitro* and *in vivo* models of ischemic stroke. Neuronal cultures were treated with NMDAR-NR1-AB, a control antibody (mGO-AB) or phosphate-buffered saline followed by NMDA exposure or oxygen-glucose deprivation (OGD). Cell death was measured by lactate dehydrogenase assay. NMDA and OGD significantly increased cell death, but NMDAR-NR1-AB did not exert neuroprotective effects *in vitro*. *In vivo*, C57BL/6J mice were subjected to middle cerebral artery occlusion (MCAo) for 45 min and treated intraperitoneally with NMDAR-NR1-AB or mGO-AB. Lesion size and neurobehavioral outcomes were assessed at 24 and 72 h and 28 days after MCAo. No differences in lesion sizes or long-term neuroprotective effects were evident at 24 h and 28 days post-MCAo. These findings underscore both the potential and limitations of targeting NMDAR-mediated excitotoxicity in ischemic stroke therapy and highlight the need for further research into the long-term efficacy of NMDAR-NR1-AB.

## 1 Introduction

Ischemic stroke remains a pre-dominant cause of long-term disability globally, with a significant rise in both incidence and prevalence rates documented from 1990 to 2021 (Virani et al., 2021; GBD 2021 Nervous System Disorders Collaborators., 2024). Currently, therapeutic interventions are largely confined to thrombolytic agents, such as recombinant tissue plasminogen activators (alteplase and tenecteplase), which offer a limited therapeutic window and present numerous contraindications (Fu et al., 2015; Alamowitch et al., 2023). Thrombectomy, as an additional critical intervention for acute stroke, has an efficacy that is also highly time-sensitive, typically requiring initiation within 6 h of symptom onset to optimize outcomes (Muir et al., 2017).

One of the critical mechanisms underlying secondary neuronal injury in ischemic stroke is excitotoxicity, primarily mediated by excessive glutamate release (Belov Kirdajova et al., 2020). This pathological release activates NMDA receptors (NMDARs), resulting in excessive calcium influx and subsequent activation of pro-apoptotic signaling cascades. Despite extensive research since 1984 into NMDAR inhibition as a potential therapeutic approach, translation into clinical success has been hindered by the inability to selectively inhibit the deleterious signaling pathways without affecting the neuroprotective functions of NMDARs (Albers et al., 1989; Gladstone et al., 2002). Additionally, adverse outcomes such as increased mortality, behavioral alterations, and neurotoxicity have been reported in various studies (Chen et al., 2008; Moore et al., 2022).

In a pivotal development in 2007, the discovery of antibodies specifically targeting the NR1 subunit of NMDARs (NMDAR-NR1-AB) in the context of NMDAR encephalitis shifted the paradigm (Dalmau et al., 2011). Subsequent investigations revealed the presence of these antibodies at lower concentrations in otherwise healthy individuals, suggesting a broader physiological significance (Hammer et al., 2014). Intriguingly, NMDAR-NR1-AB seropositivity has been correlated with reduced lesion sizes in ischemic stroke patients, although these individuals also exhibited higher incidences of depression and recurrent vascular events within 3 y post-stroke (Busse et al., 2014).

Pre-clinical studies further corroborate these findings, demonstrating substantial reductions in lesion size following immunization against the NR1 subunit in rodent models (During et al., 2000). However, the therapeutic efficacy of monoclonal NMDAR-NR1-AB treatments has yet to be established. While the blood-brain barrier (BBB) generally restricts antibody penetration, ischemic events that compromise BBB integrity may permit localized antibody entry and consequent NMDAR inhibition (Dalmau et al., 2011). Pre-existing data indicate that these antibodies can persist and remain active within the CNS, suggesting a potential advantage over traditional NMDAR antagonists (Zerche et al., 2015).

In addition, NMDAR activation has distinct acute and late effects (Paoletti et al., 2013). In the acute phase, excessive NMDAR activation causes the explained excitotoxicity by increasing calcium influx, leading to neuronal damage. In the late phase, however, NMDAR activation supports motor recovery and neuroplasticity, which is essential for long-term healing and new neuronal connections (Hardingham and Bading, 2010). The therapeutic challenge is to reduce excitotoxicity in the acute phase without compromising the beneficial effects of the late phase.

We therefore hypothesize that during the acute or subacute phase, antibodies enter the brain through a leaky BBB and block excessive NMDAR activity, thereby providing neuroprotection. As the BBB heals, the antibodies degrade, allowing the NMDARs to resume normal function, supporting recovery and neuroplasticity in the late phase. In addition, we propose that early intervention with NMDAR-NR1 antibodies during the acute phase may lead to lasting improvements by mitigating excitotoxic damage shortly after the onset of ischemia which could contribute to better long-term functional recovery. In this study, we define the acute phase of ischemic stroke as the initial period up to approximately 24–72 h following vessel occlusion during which pathological processes such as excitotoxicity, energy depletion, oxidative stress and BBB disruption are most prominent. This definition aligns with current guidelines which describe the acute phase as the period from the first hours up to 72 h after onset when timely intervention is critical in reducing irreversible brain damage (Powers et al., 2018).

This study aims to elucidate the neuroprotective potential of a novel monoclonal NMDAR-NR1 antibody by assessing its impact on lesion size and long-term functional outcomes in a murine model of ischemic stroke. We hypothesize that these circulating antibodies, upon BBB disruption, can mitigate NMDA-mediated excitotoxic damage, thereby offering a neuroprotective strategy.

Therefore, our study introduces a novel recombinant monoclonal antibody targeting the NR1 subunit of the NMDA receptor. Unlike previous vaccination-based methods, our recombinant antibody offers higher specificity and consistency, addressing the variation often seen in polyclonal immune responses. In addition, the intraperitoneal administration used in our study further differentiates it from previous approaches, which may affect BBB interaction and tissue bioavailability. This novel combination of a highly specific antibody and an alternative route of administration offers the potential for more effective and controlled modulation of NMDA receptor-mediated neuroprotection.

## 2 Materials and methods

### 2.1 NMDAR-NR1 antibodies

The human monoclonal NR1 IgG antibody 003-102 (NMDAR-NR1-AB) was previously obtained from a plasma cell isolated from cerebrospinal fluid (CSF) of a patient with autoimmune encephalitis (Kreye et al., 2016). A strong inhibitory effect on NMDAR-mediated ionic currents and a reproduction of AB-induced decrease of NMDAR-concentrations in hippocampal neuronal cultures have been demonstrated (Kreye et al., 2016). Whole-cell patch clamp recordings revealed a 46% reduction in NMDAR-mediated currents in hippocampal neurons treated with the NR1-IgG-AB compared to control [119 ± 15 pA vs. 220 ± 61 pA, *P* = 0.016, (Kreye et al., 2016)]. Additionally, immunohistochemistry showed that the antibodies effectively targeted *in vivo* epitopes in the mouse brain, binding strongly to the hippocampal neuropil and cerebellar granule cells (Kreye et al., 2016; Jurek et al., 2019). Electrophysiological analysis confirmed that these antibodies disrupted synaptic NMDAR currents and morphology, while calcium imaging experiments showed significantly reduced NMDA-induced calcium influx. Given this validation and the identical AB used in our study, produced under identical conditions, we did not repeat the immunohistochemical or electrophysiological experiments as the functionality of the antibodies had already been established (Kreye et al., 2016). As a negative control, we used mGO53 (mGO-AB), a non-brain-reactive human AB previously described by Wardemann et al. (2003). It is commonly used as an isotype control antibody in experimental animal studies (Kuchling et al., 2023; Yin et al., 2024). For recombinant AB production, HEK293T cells were transiently transfection with paired expression vectors encoding for the AB’s heavy and light chain. AB was purified from cell culture supernatants and concentration determined using an anti-human IgG ELISA, all as previously described (Kreye et al., 2016).

### 2.2 *In vitro* experiments

Primary neuronal cultures of mouse cerebral cortex were derived from fetal C57BL/6N mice at embryonic day E15 as previously described (Boujon et al., 2019). In brief, dissected cerebral cortical tissue was incubated at 37°C for 15 min in trypsin/EDTA (0.05/0.02% w/v in phosphate buffered saline, PBS, PanBiotech), followed by two wash steps with PBS w/o Ca/Mg (PBS w/o) and dissociation medium (modified Eagle’s medium with 10% fetal calf serum, 10 mM HEPES, 44 mM glucose, 100 U/mL penicillin/streptomycin, 2 mM l-glutamine). The resulting material was mechanically dispersed in the dissociation medium, pelleted by centrifugation (225 g) for 2 min and resuspended in starter medium (Neurobasal Medium and Supplement B27 [Gibco]; 100 U/mL penicillin/streptomycin; 0.5 mM l-glutamine; 25 μM glutamate). Plates of 24 wells were pre-incubated at 4°C with poly-l-lysine (5 μg/mL in PBS; Biochrom) overnight. After rinsing with PBS with Ca/Mg and incubation with collagen medium (modified Eagle’s medium with Earle’s salts, 5% fetal calf serum, 10 mM HEPES, 100 U/mL penicillin/streptomycin, 3% w/v collagen G; Biochrom) the culture cells were plated out in 600 μL starter medium at a density of 300.000 cells/well. Cultures were fed with cultivation medium (starter medium without glutamate) on the 3rd *in vitro* day by replacing 30% of the medium.

#### 2.2.1 NMDA-stimulation

On day 9 of the experiment the cultivation medium was reduced to 400 μL and the cultures received treatment with either the NMDAR-NR1-AB or the non-reactive mGO-AB. A third section of wells was not pre-treated with any of the antibodies and was considered the no treatment group (noT). Following a 6-h incubation period the cultures were exposed to 100 μM NMDA at a concentration of 1 μg/mL per well. After incubation for 20 h cell death was evaluated using lactate dehydrogenase assay described below.

#### 2.2.2 Oxygen glucose deprivation

On day 7 after plating in a second experiment with the same steps of cell cultivation as described above, the medium of each well was reduced to 400 μL and collected as basal medium. The neurons were pre-treated with NMDAR-NR1-AB or the control mGO-AB in dosages of 0.1, 1 and 10 μg/mL antibody per mL Medium. A third group received the same amount of PBS (no treatment group = noT), to exclude effects due to additional liquid inoculation. For preparation of the collected medium, 30% of fresh medium with PBS or with the antibodies were added in the appropriate final concentration and the medium was stored at 4°C overnight. Oxygen–glucose deprivation (OGD) was performed 12 h later to induce Ischemic-like stress as described previously (Goldberg and Choi, 1993; Gertz et al., 2012). In brief, after removing the remaining medium and adding balanced salt solution without glucose (BSS0) the cultures were subjected to 180 min OGD in a humidified, temperature-controlled (36 ± 0.5°C) hypoxic chamber (0.5% O_2_; INVIVO2 400, Ruskin Life Sciences). The BSS contained 1,160 mM sodium chloride, 54 mM potassium chloride, 1M calcium chloride, 8 mM magnesium sulfate, 10 mM monobasic sodium phosphate. 1M HEPES buffer (BioXtra, BioReagent), 30 mM glycine and 1M sodium bicarbonate diluted in distilled water. In the control condition, the cultures were incubated in a normoxic atmosphere with 5% CO_2_ for the same duration as in the OGD condition, after replacing the medium with BSS with 20 mM d-glucose (BSS20). In our experiment, the BSS0 did not contain any glucose. Although d-glucose is metabolizable, its presence in the control medium serves as a comparison to OGD conditions, ensuring that any observed effects in the treatment group are due to the lack of glucose and oxygen. After OGD, the balanced salt solution was exchanged with the prepared collected medium and the cultures were further incubated for 12 h in a standard cell culture atmosphere for 20 h.

#### 2.2.3 Lactate dehydrogenase assay

An assay, measuring the activity of LDH in culture medium was used to evaluate neuronal injury after NMDA stimulation and OGD, as previously described (Koh and Choi, 1987; Freyer and Harms, 2017). Briefly, concentrations of LDH were determined using a kinetic photometric assay 20 h after injury induction for both *in vitro* experiments. Fifty microliters of culture media were pipetted into 96-well plates and mixed with 200 μL of β-nicotinamide adenine dinucleotide solution (0.15 mg/mL in 1 × LDH buffer). Measurement was started immediately after addition of the reaction substrate pyruvate (50 μL of 22.7 mm pyruvate solution). Optical density was measured at 340 nm using a microplate reader (Dynex Technologies, model: MRX Microplate Reader), by 10 counts with 30 s intervals, followed by calculation of results using an LDH standard (Greiner; DiaSys). The maximum release of LDH was achieved by 20 min cell lysis with Triton X-100 as described recently, and data were normalized to these measurements (Harms et al., 2001; Freyer and Harms, 2017).

### 2.3 Animal experiments

#### 2.3.1 Ethical guidelines

All experimental procedures were approved by the “Landesamt für Gesundheit und Soziales Berlin” under the registration number G0261/17 and were in accordance with national and institutional guidelines for the care and use of laboratory animals and with directive 2010/63/EU of the European Parliament and of the Council of 22 September 2010. All experiments were also performed in accordance with the ARRIVE 2.0 guidelines (Percie du Sert et al., 2020).

#### 2.3.2 Experimental design

We performed two consecutive *in vivo* experiments with a total of 86 male 10 weeks old C57BL/6J mice. In a first experiment, we intended to determine the optimal antibody dosage for the following experiments. We performed MCAo on 50 C57BL/6J mice which received either mGO-AB or NMDAR-NR1-AB 24 h prior to the procedure on day 7 upon arrival after habituation to our facilities. The day the mice underwent MCAo marks day 1 of our dose-finding experiments. To determine the optimal antibody dosage, ischemic lesion volume on Magnetic Resonance Imaging (MRI) was assessed 24 h after MCAO as the primary outcome parameter.

In previous studies the dose to reach the brain tissue could already be determined to be around 240 μg per adult mouse, corresponding to approximately 10 mg/kg bodyweight [BW, (Jurek et al., 2019)]. However, these studies did not investigate the dynamics of BBB permeability after cerebral ischemia. Additionally, no possible neuroprotective effect after cerebral ischemia was analyzed. Therefore, the described dosage only served as a reference in our experiments. Thus, to determine the optimal dosage of the antibodies we used the reference dose of 10 mg/kg BW and two other doses, each reduced by the factor of 10 (0.1 and 1 mg/kg BW, respectively). To avoid the triggering of pro-apoptotic pathways, we did not use higher doses of the antibody.

**FIGURE 1:**
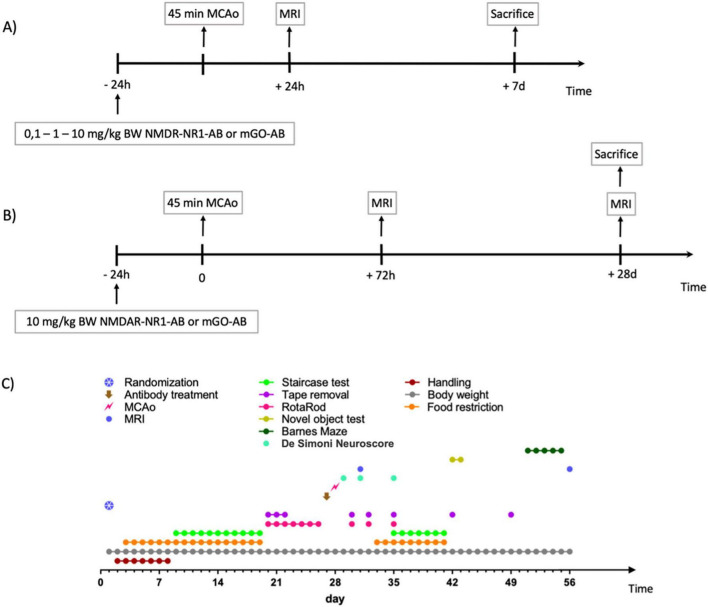
Experimental protocols. **(A)** Dose finding experiment. Animals were either treated with 0.1 (*n* = 8), 1 (*n* = 10) or 10 mg/kg (*n* = 12) bodyweight (BW) NMDAR-NR1-ab or 1, or 10 mg/kg (*n* = 10) BW control ab (mGO-AB) intraperitoneally. MCAo was performed 24 h after AB-administration followed by MRI at 24 h after MCAo. Mice were sacrificed at day 7 after MCAo. **(B)** Long-term functional study: Timeline. Administration of 10 mg/kg BW NMDAR-NR1-AB (*n* = 18) or mGO-AB (*n* = 18) intraperitoneally. MCAo was performed 24 h after AB-administration after a handling period of 28 days, followed by MRI 72 h after MCAo. Animals then underwent a series of different functional assessments, which are fully described in the methods section and seen on **(C)**. At day 28 post-MCAo MRI for atrophy determination was performed, followed by sacrifice of the animals. **(C)** Long-term functional study: Experimental set-up. Each behavioral test, indicated by different colored dots as explained in the legend, was performed at different times during the study. MCAo was induced on day 28 after the handling period, and animals were euthanized on day 56 after arrival (day 28 after MCAo).

In a second experiment, we assessed the long-term functional effects of NMDAR-NR1-AB on behavioral outcome parameters. Effects were studied in 36 mice over a 56-day period (Figure 1). Focal cerebral ischemia was induced with 45-min MCAo on day 28, with mice aged 14 weeks. At the time of sacrifice, the animals were decapitated under deep isoflurane anesthesia, and the brain was extracted after opening the skull. The samples were snap-frozen in methyl butane (Carl Roth) at −78°C and temporarily stored at −80°C until further use. All experiments were designed according to the STAIR criteria (Fisher et al., 2009) and conducted by the same female experimenter blinded to treatment allocation.

#### 2.3.3 Animals

A total of 86 male C57BL/6J mice (10 weeks old, mean weight 25 ± 2 g) were purchased from Charles River Germany (Table 1). The animals were housed SPF like in groups of 3–5 per cage with a standard light-dark cycle (12 :12 h) and *ad libitum* access to food and water. Animals were rested for 7 days upon arrival to the laboratory.

**TABLE 1:** Animals used in the study.

Experiment	Treatment	Dose (mg/kg/BW)	Included (N)	Total (N)	Died (N)	Excluded (N)	T2 edema corrected lesion volume (mm^3^)
**Dose finding**	mGO-AB	1	10	10	0	0	44.3
		10	9	10	2	1	45.9
	NMDAR-NR1-AB	0.1	8	8	2	0	44.6
		1	12	12	1	0	39
		10	10	10	1	0	32.4
**Long-term outcome**							**72 h post MCAo**
	mGO-AB	10	17	18	0	1	18.7
	NMDAR-NR1-AB	10	17	18	1	1	17.7
							**Brain atrophy 28 d post MCAo**
	mGO-AB	10	17	18	0	1	−9.67
	NMDAR-NR1-AB	10	17	28	1	1	−11.9
**Total**			83	86	7	3	

One animal was excluded from all subsequent analyses due to premature euthanasia necessitated by its deteriorating condition. In addition, two animals, one from the long-term experiment and one from the dose-finding experiment, had no detectable MRI lesions at 72 and 24 h after MCAo, respectively, and were therefore excluded from further analysis.

#### 2.3.4 Randomization, blinding, exclusion criteria

For randomization, animals were numbered, and operation order was randomly assigned using (Research Randomizer, Version 4.0, Urbaniak, G. C., & Plous, S.). For all protocols and outcome, assessments were performed by observers blinded to previous treatments. Exclusion criteria were a weight loss of > 10% within 3 days, including the inability to eat and move because of poor condition and a negative MRI for lesion size detection.

#### 2.3.5 Antibody production, storage and administration

Antibody production was performed culturing human embryonic kidney cells (HEK293T) as described by Kreye et al. (2016). The harvested supernatant after three and 5 days was used for antibody purification. ELISA was performed to determine the IgG concentrations after IgG extraction. To prove NR1 reactivity beforehand, NR1 DNA was transferred on HEK293T cells. The cells were then handled in a previously described way and incubated with the monoclonal human antibodies overnight at 4°C (Kreye et al., 2016). Double-labeling images were generated for immunohistochemical quantification. The antibody was stored at 4°C. Intraperitoneal administration of either 0.1, 1 or 10 mg/kg body weight antibody was performed 24 h before MCAo. Again, we used mGO-AB as a control as previously described (Wardemann et al., 2003). To reduce animal numbers, we only included groups treated with mGO-AB at doses of 1, and 10 mg/kg body weight, as we expected potential effects of the non-binding antibody primarily in higher concentrations.

#### 2.3.6 Model of middle cerebral artery occlusion in mice

All experimental procedures were conducted by a trained experimenter following published standard operating procedures as implemented in our laboratory (Engel et al., 2011). As described previously, a transient filament MCAo model was generated (Engel et al., 2011). Mice were anesthetized using 2.5% (vol/vol) isoflurane for induction and 1.5–2% (vol/vol) isoflurane for maintenance, mixed in both cases with 30% O_2_ and ∼68% N_2_O. After permanent occlusion of the ipsilateral external carotid and the common carotid artery, cerebral ischemia was induced by introducing a 7–0 silicon-rubber-coated MCAo suture with a coating length of 9–10 mm (monofilament 7019910PK5Re, Doccol Corp., Sharon MA, United States) into the left internal carotid artery and advancing it up to the anterior cerebral artery, thereby occluding the middle cerebral artery. The filament was withdrawn after an occlusion time of 45 min. The 45-min occlusion time was selected based on established protocols demonstrating reliable infarct induction with low variability and acceptable survival in rodents and especially in C57BL/6 mice (Bederson et al., 1986; Engel et al., 2011; Knauss et al., 2020).

Rectal temperature was maintained at 37.5 ± 0.5°C throughout the experiment using a feedback-controlled heating pad with a rectal probe (Fine Science Tools GmbH, Heidelberg, Germany). During MCAo and for at least 2 h after reperfusion, animals were allowed to recover in a heated cage (Peco Services, Cumbria, United Kingdom). Afterward, they were transferred to the home cage with access to soft food. The animals were treated with subcutaneous physiological saline injections for 1 week to prevent postsurgical dehydration.

#### 2.3.7 Magnetic resonance imaging

For Magnetic Resonance Imaging (MRI) the animals were anesthetized under 1.5–2% isoflurane with a vaporizer in a mixture of 30% O_2_/70% N_2_O and placed prone and head fixed on a heated circulating water pad to assure a stable body temperature of 37 ± 0.5°C during scanning. Respiratory rate was monitored constantly (Small Animal Monitoring & Gating System, SA Instruments, Stony Brook, New York, United States). Images were acquired on a 7-Tesla rodent scanner running Paravision 5.1 software (Pharmascan 70/16, Bruker, Ettlingen, Germany). A 20 mm quadrature volume resonator (RAPID, Biomedical, Rimpar, Germany) was used for imaging. Scout images were obtained after tuning and adjustment of the volume resonator. For T2 – weighted anatomical imaging the anterior commissure was used as a landmark in rostral-caudal direction, whereas the trachea was used as a landmark in the other directions, to ensure similar geometry of all scans across the mice. T2-weighted images were generated using a 2D turbo spin-echo sequence (2D RARE) with the following parameters: Repetition time (TR) = 4,200 ms, echo time (TE) = 36 ms, echo spacing (ΔTE) = 12 ms, RARE factor = 8, 4 averages, 32 contiguous axial slices with a slice thickness of 0.5 mm, field of view (FOV) = 25.6 × 25.6 mm^2^, matrix size MTX = 256 × 256, bandwidth BW = 46,875 Hz, and total acquisition time TA = 6 min 43 s). Volumetry was performed using Analyze 10.0 software (AnalyzeDirect, Overland Park, KS), after conversion of the raw data to ANALYZE format in NIH Image J.^1^ Hyperintense areas of ischemic tissue in the T2-weighted images were assigned with a region of interest tool. Lesion volume was edema-corrected by non-linear registration to a standard brain as described before (Koch et al., 2019).

#### 2.3.8 Immunofluorescence staining

Immunofluorescence imaging was performed, as described previously (Kreye et al., 2021). Cryosections were thawed, rinsed with PBS, then blocked using a PBS solution supplemented with 2% BSA (Roth) and 5% Normal Goat Serum (Abcam) for 1 h at room temperature. An Alexa Fluor 488–conjugated goat anti-human IgG antibody (Dianova; #109-545-003) was diluted in blocking solution as above (1:1,000) and added for 2 h at room temperature, before washing three times. Images were recorded under an inverted fluorescence microscope (Olympus CKX41, Leica DMI6000).

#### 2.3.9 Long term functional study

##### 2.3.9.1 Handling

We followed an extended handling protocol based on other frequently used methods (Hurst and West, 2010). The mice were acclimated for 7 days with food and water *ad libitum*. On day 1, the experimenter placed a gloved hand in the cage for 10 min, allowing free exploration, followed by a 30-min rest and repetition. On day 2, mice approached the hand voluntarily for 10 min, followed by cupping and sitting or walking on the experimenter’s hand for 2 min. For the next 5 days, hands were lightly moved in the cage for 10 min to encourage voluntary interaction. The animals were never picked up by their tails.

##### 2.3.9.2 Modified De Simoni Neuroscore

A modified De Simoni Neuroscore (De Simoni et al., 2003; Donath et al., 2016), evaluating general behavioral alterations as well as focal motor, sensory, reflex and balance impairments was recorded at days 1, 3, and 7 following MCAo. In brief, general health and behavioral alterations and specific focal deficits were scored separately and subsequently added to form a summation score. The assessment consists of 12 test items, which are described in detail in Supplementary Table 1.

##### 2.3.9.3 Staircase test

The Staircase test was performed daily for 11 days before and up to 21 days after MCAo for conditioning and testing skilled forelimb motor function, as described previously (Montoya et al., 1991; Knab et al., 2023). For this test only, mice were subjected to a food restriction phase of 12 h overnight to enhance the motivation and need for food for the following test day. During testing, they were placed in a Plexiglas box with a baited double-sided staircase, offering 20 mg sucrose pellets on each of 8 steps. Each mouse was required to ascend onto a central base with one of the staircases on either side, designed in a way that only the ipsilateral paw can reach a given staircase. The result was measured as the number of pellets grabbed with each front paw. In the training phase animals were tested three times a day for 30 min per session. The results of the last 4 days of the training period were used as a baseline. In the period after MCAo there was only one run of 20 min each day.

##### 2.3.9.4 Rotarod

The Rotarod, a test of balance and coordination, was performed in a training phase 7 days before MCAO and at days 2, 7, and 14 following MCAo as implemented in our laboratory (Gertz et al., 2012). Between sessions the mice were allowed to rest for 15 min. The result was measured in seconds (s) until the animal fell. The best out of three runs per time point was used for statistical analysis.

##### 2.3.9.5 Adhesive tape removal

The Adhesive tape removal, a test for sensory and motoric forelimb function (Bouet et al., 2009), is a sufficient tool to highlight the impairment of the right paw after MCAo. The test was performed 3, 5, 7, 14, and 21 days after MCAo. To provide baseline values, mice were trained for 3 days (3 sessions/day) at −8 to −6 days prior to MCAo. A red adhesive tape was applied on the hairless part of the right forepaw and the time to remove it (cutoff: 120 s) was recorded.

##### 2.3.9.6 Novel object recognition

To assess the effect of NMDAR antibody treatment on exploration and memory, mice underwent the Novel Object Recognition (NOR) (Ennaceur, 2010) on day 14 post-MCAo. The objective of this test was not to assess a potential beneficial effect of the antibodies on post-stroke cognitive outcomes which was out of scope of our study. After 10 min of habituation in an empty cage, two identical objects were placed equidistantly. Mice explored for 10 min (trial 1). The right object was then replaced with a novel object of similar size but different shape. After a 2-h delay, mice explored again for 5 min (trial 2). Parameters measured included time spent and number of visits to each object. The discrimination ratio was calculated as [right familiar/(right familiar + left familiar)] and [right novel/(right novel + left familiar)]. This test was included to determine if antibody treatment alone influenced exploratory behavior or memory function.

##### 2.3.9.7 Barnes maze

For learning and memory, the animals were tested in a Barnes maze, which consisted of a white circular platform with 20 holes, as implemented in our laboratories (Jurek et al., 2019). An escape box was hidden under 1 of the 20 holes and orientation clues were placed around the platform on the wall. Wrong nose dips and latency to the target box were recorded. The animals were trained for 4 days, each day containing of 4 trials with a cutoff of 180 s. The fifth day was the testing day with a cutoff of 90 s and only one trial.

### 2.4 Statistics

Statistical evaluation was performed using Prism8 (GraphPad Software, San Diego, CA). Data are presented as mean ± standard deviation (SD). Statistical analyses for comparison between two groups included Student’s *t*-test for independent samples, whereas differences between more than two groups were assessed using one-way, two-way ANOVA or a mixed linear model depending on the test requirements and Tukey’s *post hoc*, Sidak correction test or a two-stage set-up method of Benjamini, Krieger and Yekutieli. We used the D’Agostino & Pearson Test to assess the normality of our data distributions. Kaplan–Meier log-rank test was conducted for survival with Bonferroni correction. Based on assumed effects of about 30% and a standard deviation of 25% we performed a sample size calculation with a power of 0.8 (1-β) and a significance level of 0.05 (α). As a result, we set the group size at *n* = 12 for the primary outcome parameter of reduction in ischemic lesion size. We did not incorporate the attrition rate into our power calculations for this study.

## 3 Results

### 3.1 NMDAR-NR1-AB treatment does not decrease neuronal cell death after NMDA stimulation or after OGD

Cell death significantly increased after 100 μM NMDA stimulation compared to controls (36.3 vs. 18.5%, *p* < 0.05, Two-way-ANOVA. However, cell death rates were similar across the 1 μg/mL NMDAR-NR1-AB, mGO-AB, and PBS-treated groups (32.5 vs. 36.6%). LDH release did not differ between groups (10 μg/mL NMDAR-NR1-AB: 36.1%; mGO-AB: 35.7%; no treatment: 39.9%). In conclusion, antibody treatment showed no neuroprotective effect. After OGD, cell death was significantly higher compared to controls (17.2 vs. 7%, *p* < 0.001), but no differences were observed between treatment groups.

### 3.2 *In vivo* dose finding

#### 3.2.1 Immunofluorescence staining

**FIGURE 2:**
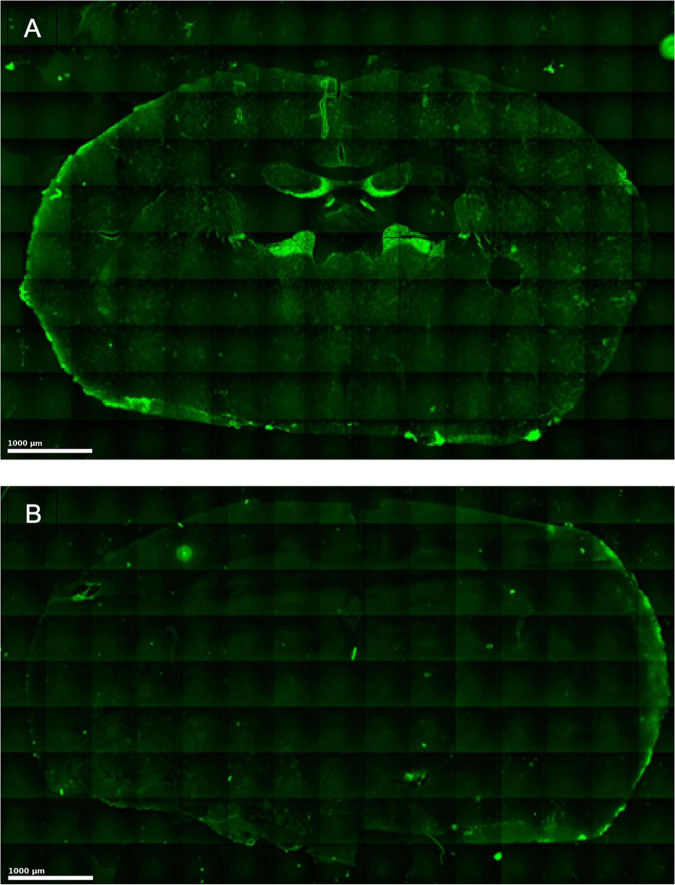
Immunofluorescence staining of human NMDAR-NR1-AB (green) on unfixed murine brain tissue. The images showing immunofluorescence staining in the treatment group **(A)** and the control group **(B)**. In the treatment group, human NMDAR-NR1 antibodies strongly bind to neuronal structures whereas the control (anti-human IgG) shows minimal to no specific binding.

Immunofluorescence staining of brain tissue cryo-sections confirmed that human IgG was diffused into and accumulated in the brains of the treated animals (Figure 2). Based on these findings and the radiological data we decided to use the highest concentration of 10 mg/kg bodyweight as a start dose for the dose-response experiment.

#### 3.2.2 No differences in lesion sizes after 24 h between the treatment groups

**FIGURE 3:**
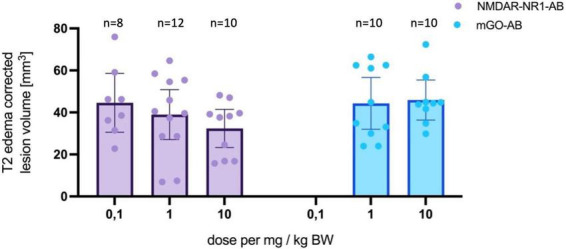
T2 edema corrected lesion volumes assessed via T2-weighted MRI in NMDAR-NR1-AB or mGO-AB treated animals 24 h after MCAo. Data are represented as mean and 95% CI as follows: Mean lesion volume NMDAR-NR1-AB 0.1 mg/kg BW dose: 44.6 mm^3^, 95% CI: 30.6, 58.5. Mean lesion volume NMDAR-NR1-AB 1 mg/kg BW: 38.9 mm^3^, 95% CI: 27.2, 50.6. Mean lesion volume of NMDAR-NR1-AB 10 mg/kg BW: 32.5 mm^3^, 95% CI: 23.3, 41.5. Mean lesion volume mGO-AB 1 mg/kg BW: 44.3 mm^3^, 95% CI: 31.9, 56.7. Mean lesion volume mGO-AB 10 mg/kg BW: 45.9 mm^3^, 95% CI: 36.4, 55.5. No significant effect between treatment groups and dose. Effect of treatment: *F*(1, 45) = 3.38, *p* = 0.073.

**FIGURE 4:**
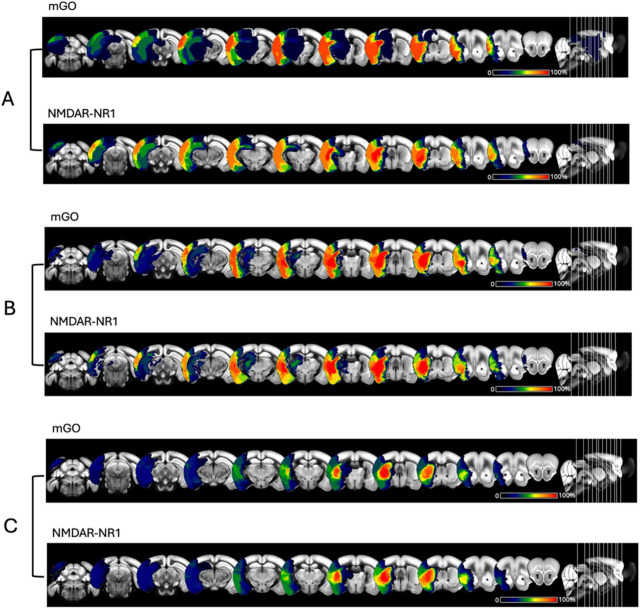
Incidence maps of MRI T2 imaging for the dose-finding **(A)**: 1 mg/kg BW, **(B)**: 10 mg/kg BW and the long-term functional study **(C)** animals for each treatment group. There is no significant difference of edema-corrected lesion sizes between the treatment groups.

Fifty animals received treatment of NMDAR-NR1-AB or mGO-AB in different doses (0.1 or 1 or 10 mg/kg BW) intraperitoneally 24 h prior to 45 min MCAo (Table 1). In animals treated with 10 mg/kg NMDAR-NR1-AB (*n* = 10), infarct volumes were reduced by 21% (34.5 mm^3^ vs. 43.6 mm^3^, *p* = 0.06, Two-Way ANOVA, mixed-effects analysis 24 h after MCAo compared to mGO-AB treated controls *n* = 10, 9 included, 1 no lesion). In animals treated with 1 mg/kg NMDAR-NR1-AB (*n* = 12), we again observed a reduction in lesion size of 19% (37.3 mm^3^ vs. 46.4 mm^3^, *p* = 0.6, Figure 3) compared to mGO-AB treated animals (*n* = 10). In addition, our results indicate that smaller lesions could be detected with increasing doses of NMDAR-NR1-AB (0.1 mg/kg BW: 44.6 mm^3^, 1 mg/kg BW: 37.3 mm^3^, 10 mg/kg BW: 34.5 mm^3^, *p* = 0.3, mixed-effect analysis, Figure 3). Incidence Maps of lesion volumes in MRI are displayed in Figure 4.

### 3.3 Long-term functional and histological outcome

#### 3.3.1 Treatment with NMDAR-NR1-ABs before MCAo does not reduce lesion volume after 72 h or brain atrophy at 28 days after MCAo

Distribution of lesion volumes at 72 h after MCAo in 10 mg/kg BW NMDAR-NR1-AB treated mice (*n* = 18) was similar to mGO-AB treated animals (*n* = 18, 17.7 mm^3^ vs. 18.7 mm^3^, 95% CI -6.65 to 2.23, *p* = 0.85, Two-tailed *t*-test). Additionally, brain atrophy measured at day 28 after MCAo did not show a significant difference between the treatment groups (NMDAR-NR1-AB -9.67 mm^3^ vs. mGO-AB -11.9 mm^3^, 95% CI −10.0 to 12.0, *p* = 0.31, Two-tailed *t*-test).

#### 3.3.2 NMDAR-NR1-AB treatment does not statistically significantly improve functional outcome assessed for up to 28 days after MCAo

A tendency toward improved performance of the NMDAR-NR1-AB treated animals compared to mGO-AB treated animals in all neurobehavioral tests was noted, although statistical significance was not achieved. Descriptively NMDAR-NR1-AB treated animals performed better in Adhesive Tape removal, Rotarod and Barnes Maze compared to mGO-AB treated animals. However, inferential statistics did not reveal significant differences between treatment groups in any of the performed functional tests. Furthermore, although no neuropsychiatric test battery was used, we observed no apparent neuropsychiatric abnormalities, such as hyperactivity, social withdrawal or refusal to complete tasks in any of the treatment groups during the testing period.

#### 3.3.3 Modified De Simoni Neuroscore

**FIGURE 5:**
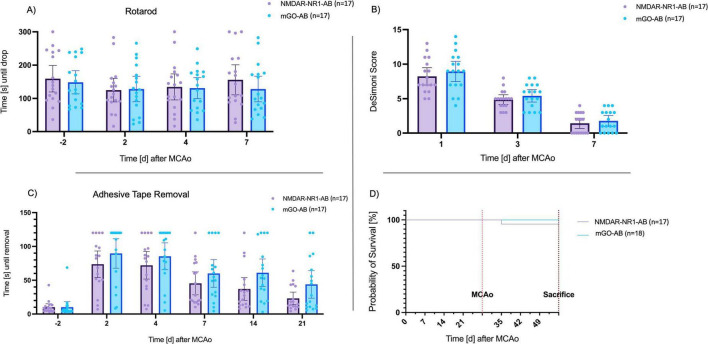
Long-term functional outcomes of neurobehavioral tests. **(A)** Time until drop from the Rotarod (s): Rotarod performance was decreased in animals after MCAo. Seven days after MCAo, mice in both groups reached baseline values. No significant differences between the treatment and control group. Data are presented as Tukey boxplot with 95% CI. Two-way repeated measures ANOVA. Effect of treatment group: *F*(1, 32)=̃ 0.2, *p* = ~ 0.63; Effect of time point: *F*(2.271, 74.95) = 1.734, *p* = ~ 0.18. **(B)** De Simoni Score. Score values decreased after MCAo. No significant differences between the treatment and control group. Data are presented as Tukey boxplot with 95% CI. Two-way repeated measures ANOVA. Effect of treatment group: *F*(1, 32) = 0.9, *p* = 0.33; Effect of time point: *F*(2, 66) = 192, *p* < 0.0001. **(C)** Remove of the adhesive tape (s): Numerical trend without statistical significance toward faster tape removal in the treatment group. Effect of treatment group: *F(*1, 32) = 2.95, *p* = 0.096; Effect of time point: *F*(3.36, 107) = 40.1, *p* < 0.001; Effect of time point x treatment group: *F*(5, 160) = 0.931, *p* < 0.001. **(D)** Kaplan–Meier curve of long-term survival of animals treated with NMDAR-NR1-AB (*n* = 18) or mGO-AB (*n* = 17): Mortality in NMDAR-NR1-AB-treated animals was 3.6% after 28 days, whereas none of the mGO-AB treated animals died. This difference was not significant (*p* = 0.31).

Score values in the modified De Simoni Neuroscore decreased significantly in both groups during time (*p* < 0.0001). However, no significant difference between the groups could be determined (day 1 post-MCAo: NMDAR-NR1-AB vs. mGO-AB: 8.24 vs. 8.94 *p* = 0.44, day 3 post-MCAo: NMDAR-NR1-AB vs. mGO-AB: 4.88 vs. 5.41 *p* = 0.33, day 7 post-MCAo NMDAR-NR1-AB vs. mGO-AB: 1.41 vs. 1.76 *p* = 0.50, multiple unpaired *t*-tests, Figure 5).

#### 3.3.4 Rotarod

Post-ischemic mice in both treatment groups remained for shorter periods on the Rotarod as compared to baseline values on day 2 after MCAo. However, time until drop after MCAo did not significantly differ compared to baseline values (*p* = 0.12). Animals in the NMDAR-NR1-AB group stayed longer on the Rotarod than the control group on day 7 after MCAo (156 s, SD 77.5 vs. 128 s, SD 67.9 s, Figure 5). However, inferential statistics did not reveal significant differences between groups (*p* = 0.63, Two-Way ANOVA).

#### 3.3.5 Adhesive tape removal

Animals of both treatment groups needed significantly longer to remove the tape after MCAo as compared to baseline values (*p* < 0.0001). The NMDAR-NR1-AB group showed a trend of shorter time needed to remove the tape compared to the mGO-AB group at every timepoint after MCAo, especially on day 14 (NMDAR-NR1-AB vs. mGO-AB: 37 s vs. 61 s, *p* = 0.062, Two-Way ANOVA, Figure 5) and day 21 (NMDAR-NR1-AB vs. mGO-AB: 23 s vs. 44 s, *p* = 0.062, Two-Way ANOVA) after MCAo.

#### 3.3.6 Staircase test

On average, animals showed less use of the right paw compared to the contralateral side determined by grip performance at baseline, prior to MCAo (right paw: 75.4% vs. left paw 87.1% of baseline values, *p* = 0.009, mixed-effect analysis). Both groups showed a recall of learned skills before MCAo when tested with their left paw after just 1 day, but neither group reached their initial skill levels with their right paw. Overall, no statistical differences were observed between the groups (*p* = 0.23).

#### 3.3.7 Novel object recognition

The time spent with each object as well as the total number of visits to each object in the NOR test, as determined by nose touches, did not differ between treatment groups. The NOR test was performed on day 14 post-MCAo without pre-exposure to the arena or objects prior to MCAO induction. The test was not designed to evaluate MCAO-induced cognitive deficits *per se* but rather aimed to identify any detrimental effects of the antibody treatment itself on exploratory behavior and memory. Importantly, no such differences between treatment groups were observed, suggesting that the antibody treatment did not independently impair cognitive function as assessed by this paradigm.

#### 3.3.8 Barnes Maze

In the Barnes Maze all animals showed a decreasing latency to the target hole as well as fewer wrong nose dips over time, suggesting a memorization during the training period. Both treatment groups showed similar training performance: NMDAR-NR1-AB vs. mGO-AB: latency 150 vs. 145.8 s and wrong nose dips. 12.9 vs. 13.5). On the test day, with a reduced exploration time of 90 s, six mice of the NMDAR-NR1-AB group and three mice of the control group made it into the target hole. However, no statistical differences in the latency to the target hole or the wrong nose dips were seen between the groups (*p* = 0.45 and *p* = 0.54, respectively).

## 4 Discussion

In this explorative study, we investigated the neuroprotective potential of a human recombinant monoclonal antibody against the NMDA receptor subunit NR1 using the MCAo mouse model of ischemic stroke and *in vitro* neuronal cultures. Previous studies have suggested significant reductions in lesion size, up to 70% in rats vaccinated with the NMDAR NR1 subunit (During et al., 2000) and up to 73% by spontaneously occurring NMDAR antibodies in humans (Zerche et al., 2015). Nevertheless, these studies did not assess the direct neuroprotective properties of purified monoclonal antibodies against the NMDAR-NR1 subunit following the STAIR guidelines for pre-clinical neuroprotective drug testing.

In our study we could not replicate previously reported neuroprotective effects in terms of lesion size reduction. This implies that the antibodies might not have a consistent unidirectional effect but may instead exhibit have different effects depending on factors as timing or dosing. Supporting this, Sperber et al. showed that NMDAR-NR1 antibodies were associated with a high rate of stroke recurrence and, at higher titers, correlated with worse outcomes after cerebral ischemia (Sperber et al., 2019; Sperber et al., 2022). Ehrenreich et al. provided a different perspective, finding a neuroprotective signal of pre-existing NMDAR-NR1-AB after stroke in patients without but not in patients with apolipoprotein E4 carrier status and a presumably chronically compromised blood-brain barrier (Zerche et al., 2015). They concluded that the relevance of serum NMDA-NR1-AB depends on their access to the brain. Their study, as well as that of Sperber et al., showed an increasing seroprevalence of these antibodies with age, reaching over 20% in individuals aged 80 y and older (Sperber et al., 2019). This finding suggests that the pathogenic effects of NMDAR1-AB may become more pronounced with age, possibly due to increased permeability of the BBB or other age-related changes in the immune system. These conflicting results highlight the complexity of the role of NMDAR-NR1 antibodies in neuroprotection *vs*. neurodegeneration. It is possible that these antibodies can reduce neural damage under certain conditions, while in other contexts, particularly in the presence of a compromised BBB, they can contribute to neural pathology. However, it is still unclear whether these antibodies are responsible for the poorer outcome or whether they are simply associated with a more severe clinical condition. In our model, mice exposed to NMDAR-NR1-antibodies showed no neuropsychiatric effects, likely because the antibodies were introduced only after MCAo, when the BBB was compromised and for a short duration. In contrast, patients with NMDAR encephalitis produce these antibodies intrathecally, leading to prolonged exposure in the CNS (Hammer et al., 2014; Ehrenreich, 2017). This prolonged exposure may explain the neuropsychiatric effects seen in patients, suggesting that the duration and extent of antibody exposure are key factors in a dose-dependent relationship. These findings are supported by a recent study by Rozenberg et al., which showed that patients with NMDA receptor encephalitis who tested positive for both NMDAR antibodies and oligoclonal bands (OCBs) experienced significantly greater impairments in attention and language and had higher CSF cell counts than patients without OCBs (Rozenberg et al., 2024). This suggests that intrathecal antibody synthesis and broader inflammatory activity, as indicated by OCBs, may contribute to neuropsychiatric manifestations in NMDA-R encephalitis. Their results underscore the importance of both the route of antibody and the extent of inflammation in determining clinical manifestations, which is consistent with our hypothesis that the effects of antibodies depend on exposure duration and the integrity of the BBB. Altogether, the discrepancy between our results and those of previous studies calls for a closer examination of the conditions under which NMDAR-NR1-AB might exert neuroprotective or pathogenic effects.

We undertook a comprehensive evaluation, including dose-response effects of NMDAR-NR1-AB pre-treatment *in vivo*, long-term functional outcomes and neuroprotective properties in two distinct cell-culture models of neuronal damage. Contrary to our hypothesis, we did not observe statistically significant short-term or long-term neuroprotective effects of the antibody *in vivo* or *in vitro*. This study is the first to assess a monoclonal antibody against the NMDA-receptor subunit NR1, which has shown strong inhibitory effects on NMDAR-mediated ion currents, in both *in vivo* and *in vitro* models of cerebral ischemia.

Descriptively, animals treated with 10 mg/kg NMDAR-NR1-AB exhibited a modest reduction in lesion size (21%) compared to controls in our dose-finding experiments (Figure 3). Additionally, we observed a dose-dependent reduction in lesion sizes within the NMDAR-NR1-AB-treated groups (Figure 3). However, formal statistical testing did not demonstrate a significant neuroprotective effect. Despite the absence of statistically significant differences in early MRI-based lesion volumes, the hypothesizes that functional recovery can occur independently of infarct size supported our rationale for assessing long-term behavioral outcomes.

For long-term outcomes, animals treated with 10 mg/kg BW NMDAR-NR1-AB 24 h before MCAo were followed up for 28 days. We observed no statistically significant differences in lesion size or behavior between treatment groups over this period. However, there was a consistent pattern of moderate improvement in three of the five behavioral tests (Adhesive Tape removal, Rotarod, and Barnes Maze) in the NMDAR-NR1-AB-treated group. Mortality rates differed slightly between NMDAR-NR1-AB and control groups (3.6 vs. 0%).

Our study utilized 86 animals, with sample size calculations based on an expected lesion size reduction of 30% and an anticipated standard deviation of 25%. Given that the efficacy of tPA in pre-clinical stroke models is around 30% (Sena et al., 2010), our study design was powered to detect a similar effect size. The probability of missing an effect of NMDAR-NR1-AB treatment of 30% or greater at a significance level of 0.05 is below 0.2. In our dose-finding experiments, we observed an average reduction in lesion volume of 20.2%. However, this difference did not reach statistical significance. A *post hoc* power analysis revealed that approximately 64 animals per group would be required to detect a moderate effect size (Cohen’s *d* = 0.5) at 80% power. The observed effect sizes in our comparisons ranged from small to moderate (Cohen’s *d* = 0.2–0.6), indicating that our study lacked the power to detect these differences statistically. The inherent variability in functional assessments necessitates exceedingly high animal numbers to detect smaller effect sizes reliably (Hetze et al., 2012).

In previous studies, rats vaccinated against the NMDA-NR1 subunit 5 months prior to MCAo showed a significant lesion size reduction of 70% (During et al., 2000). Unlike classical pharmacological NMDAR blockade, antibodies formed through vaccination selectively enter the brain tissue after BBB breakdown during ischemia, potentially avoiding adverse behavioral effects observed with NMDAR blockade. Vaccination-induced antibodies target various epitopes of the NR1 subunit, but the specific epitope responsible for neuroprotection remains unidentified (During et al., 2000). Our study utilized a specific monoclonal antibody known to selectively interrupt NMDAR-NR1 signaling *in vitro* by receptor internalization (Kreye et al., 2016). Effective penetration of NMDAR-NR1-AB through the BBB and its presence in brain parenchyma post-intraperitoneal injection has been previously confirmed and was reconfirmed in this study using immunofluorescence staining (Jurek et al., 2019).

Despite these confirmations, post-ischemic dynamics of NMDAR distribution and function may have influenced the antibody’s activity and efficacy (Hetze et al., 2012). Neutralizing antibodies against human IgG in our mouse model could have further diminished the antibody’s effects on NMDAR signaling (Howells et al., 2014). *In vitro* tests with neuronal cell cultures pre-treated with various doses of NMDAR-NR1-AB, followed by NMDA stimulation or OGD, showed no significant differences in cell death compared to controls. These findings align with clinical data indicating no neuroprotective effect of NMDAR-NR1-AB in ischemic stroke, with high titers associated with poorer functional and cognitive outcomes.

Our initial hypothesis was predicated on the assumption that acute-phase intervention with NMDAR-NR1 antibodies could confer lasting improvements by reducing excitotoxic damage immediately after ischemic onset, thereby indirectly supporting improved long-term functional outcomes. Future research should therefore focus on understanding the specific conditions under which NMDAR-NR1-AB provide either protective or adverse effects. This could include exploring the role of antibody titers, BBB integrity, age-related changes, and the timing of AB presence relative to neural injury. Only with a more detailed understanding of these variables can we fully evaluate the potential of NMDAR-NR1-AB in the clinical setting.

### 4.1 Limitations

Our study has several limitations. First, we did not examine possible pre-exposure of animals to NMDAR-NR1-AB upon arrival, nor did we assess the characteristics of the mice’s BBB before MCAo. Zerche et al. (2015) demonstrated adverse effects in patients with a leaky BBB, characterized by APOE4-carrier status, indicating the necessity of intact BBB for neuroprotection. The BBB composition differs between mice and humans, with mice having a leakier BBB due to higher proportions and complexity of neocortical astrocytes (Oberheim et al., 2006; Lui et al., 2011). Pre-existing antibodies and reduced BBB stability could have sensitized the brain to antibodies post-MCAo, reducing their neuroprotective properties in our study.

Second, our study only included young male mice, and BBB properties might differ in aged mice, possibly altering antibody effects. However, no NMDA-blocking specific side effects were observed in our behavioral analysis.

Third, the optimal antibody concentration in brain parenchyma for a robust effect is unknown. We based our doses on previous studies, which suggested 10 mg/kg BW for passive immunization in mice (Jurek et al., 2019). Considering the BBB breakdown post-MCAo, we tested doses reduced by a factor of 10 (0.1 and 1 mg/kg BW), but higher doses might have been more effective. We avoided higher doses due to potential neuropsychiatric impairments caused by NMDAR autoimmunity. Jurek et al. reported increased mortality and reduced brain volumes in neonates treated with higher NMDAR-NR1-AB doses, suggesting potential toxicity (Jurek et al., 2019). Additionally, we did not compare different routes of administration, such as intravenous or vaccination-based delivery, which may influence antibody bioavailability and therapeutic efficacy. Future studies should investigate whether alternative delivery methods improve BBB penetration and outcomes in the MCAO model.

Fourth, targeting NMDARs in acute ischemia is complicated by their dichotomous functions, based on localization (synaptic vs. extra synaptic) and subunit composition (Parsons and Raymond, 2014). While NMDAR blockade might suppress excitotoxicity, it could inhibit recovery in the chronic phase. Understanding the duration of antibody activity at binding sites is crucial, as prolonged activity might explain the moderate lesion size reduction observed in acute MRI but not in long-term studies. In addition to this, our study lacks direct evidence of antibody persistence and degradation kinetics in the brain parenchyma during the acute and subacute phases. However, recent literature suggests that the half-life of IgG-antibodies is about 9.5 days (Unverdorben et al., 2016). Future studies should include techniques such as radiolabeling or immunohistochemistry to follow the time dynamics of antibody bioavailability in the brain. Despite these complexities, no epileptic seizures or memorization problems were observed in long-term behavioral analyses, indicating appropriate learning responses post-MCAo.

However, in this study, the absence of significant cognitive deficits in the NOR test at day 14 post-MCAO may reflect a limitation of the MCAO model in inducing detectable impairments in this specific paradigm, rather than a lack of effect from the antibody treatment. Without an early post-stroke assessment to establish baseline cognitive deficits, it remains unclear whether the observed results are due to the treatment’s ineffectiveness or the model’s limitations.

Future studies should incorporate baseline assessments or alternative paradigms to better evaluate post-stroke cognitive outcomes.

Fifth, another limitation of our study is the pre-treatment design, which does not directly reflect a clinical therapeutic setting. We administered NMDAR-NR1-AB 24 h before MCAO to ensure systemic availability at the time of vessel occlusion and to exploit the transient BBB disruption during acute ischemia, facilitating CNS penetration. This strategy allowed us to assess the potential neuroprotective effects of the antibodies under conditions of acute excitotoxic stress. Additionally, given that approximately 10% of elderly individuals have circulating NMDA receptor antibodies (IgA/IgM isotypes) (Ehrenreich, 2017; Sperber et al., 2019), our design may also reflect a situation of pre-existing antibody presence at stroke onset, which remains under debate regarding its impact on ischemic vulnerability or protection.

## 5 Conclusion

In conclusion our data indicate a moderate lesion size reduction 24 h post-MCAo and better performance in most long-term behavioral tests after administration of NMDAR-NR1-AB 24 h prior to MCAo. However, these improvements did not reach statistical significance, so a clear neuroprotective effect was not demonstrated in this study. Nonetheless, antibody-based neuromodulation remains a promising approach to potentially mitigate excitotoxicity and provide neuroprotection in acute stroke. Further studies are warranted to explore its full potential in clinical settings of cerebral ischemia.

## Data Availability

The raw data supporting the conclusions of this article will be made available by the authors, without undue reservation.

## Footnotes

1
https://imagej.net/ij/
