# Supplementary material for: Intraperitoneal administration of NMDA-Subunit NR1-receptor antibodies does not improve long-term outcome in a murine MCAo-stroke model
Source: Front Neurosci. 2025 Jul 7;19:1614924. doi: 10.3389/fnins.2025.1614924 (PMC12277321; doi:10.3389/fnins.2025.1614924)
Supplement: Supplementary file 1 [file Supplementary_file_1.docx]

Supplementary Material

# Supplementary Figures


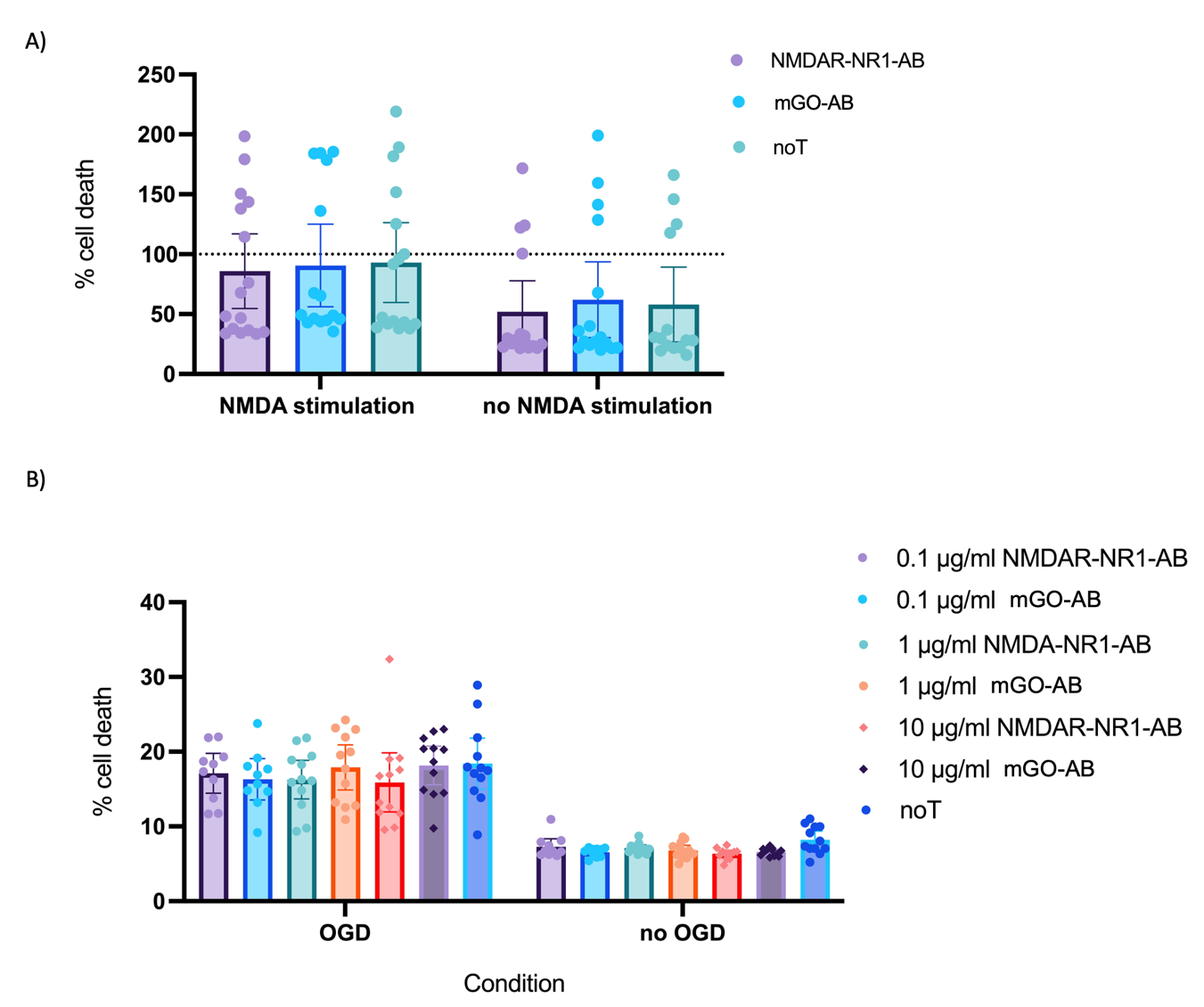


**Supplementary Figure 1.** Outcomes of in-vitro experiments.

A) NMDA stimulation: Percentage of cell death for the 1µg/ml NMDAR-NR1-AB or mGO-AB treatment group after NMDA stimulation and without NMDA stimulation. NMDA stimulation increased cell death significantly, p = 0.0033. Data are represented in mean and CI (95%). Effect of condition (NMDA vs. no NMDA): F(1, 15) = 12.2, p = 0.0033; Effect of treatment: F(2, 30) = 2.38, p = 0.11; Effect of condition x treatment: F(2, 27) = 0.5, p = 0.61.

B) Oxygen glucose deprivation: Means of cell death displayed in % for the 0.1, 1µg/ml and 10µg/ml NMDAR-NR1-AB or mGO-AB treatment group, as well as the noT group after OGD vs. no OGD (n = 12: 5 different experiments, 2 wells per experiment and treatment, for each treatment condition 2 extra wells in one experiment except 0.1µg/ml). Data are represented in mean and CI (95%): OGD lead to a significant increase in cell death. No significant difference between the treatment groups. Effect of condition: F(1, 22) = 332, p <0.001; Effect of treatment: F(3.88, 80.2) = 1.09, p = 0.37; Effect of condition x treatment: F(6, 124) = 0.3, p = 0.88.

**
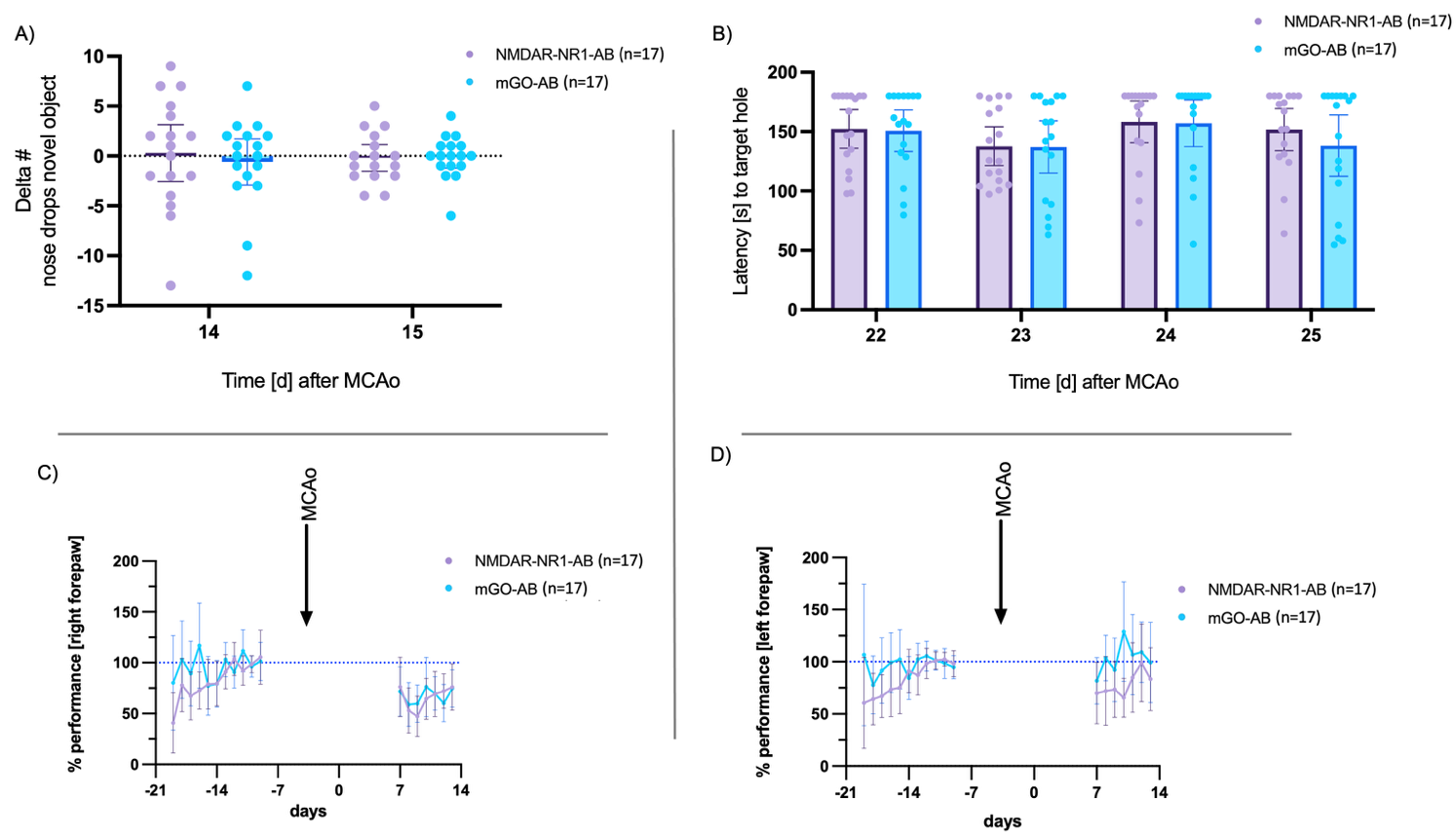
**

**Supplementary Figure 2**. Long-term functional tests.

A) In NOR the nose drops to the novel object did not differ between treatment groups from day 14 to day 14 post-MCAo. B) In the Barnes Maze, during training period (day 22-25 post-MCAO), the mean latency to the target whole was comparable in both groups (NMDAR-NR1-AB 150 s vs. mGO-AB 145.8 s). C) and D) During training period (21 to 7 days prior to MCAo) baseline values for Staircase test performance were determined (100% performance) for each forepaw. Post-MCAo mice of both groups showed better performance with their left forepaw. mGO-treated animals reached baseline values with their left forepaw after being subjected to the test apparatus for only one day, at day 8 post-MCAo. We did not observe any statistically significant differences in the staircase test.

**2 Supplementary Tables**

**Supplementary Table 1.** Assessments and evaluation criteria included in the modified De Simoni neuroscore**.**

|  | **General vs. Focal** | **Instruction** | **Score = 0** | **Score = 1** | **Score = 2** | **Score = 3** | **Score = 4** | **Summative score** |
| --- | --- | --- | --- | --- | --- | --- | --- | --- |
| Hair | General | Observation on open tabletop without interference | Hair neat and clean. | Lack of grooming, piloerection and dirt on the fur around nose and ear. | Lack of grooming, piloerection, and dirty coat beyond the nose and eyes. |  |  | 0-2 |
| Ears | General | Observation at the beginning without interference and then stimulation by clicking the tongue. | Normal. Ears are set back and to the side. They react to noise by straightening up. | Stretched laterally but not behind (one or both). They react to noise. | Same as 1. They do not react to noise. |  |  | 0-2 |
| Eyes | General | Observation on open tabletop without interference | Open and clear (no discharge). | Open and marked with milky white mucus. | Open and marked with milky dark mucus. | Eyes clotted (one or both sides). | Eyes closed. | 0-4 |
| Posture | General | Mouse placed on palm of hand, gently rocking to observe stability | The mouse stands upright on four legs with its back parallel to the palm of the hand. During the rocking motion, it uses its limbs to stabilize itself. | Hunched posture.During rocking motion, it lowers its body instead of using its limbs for stability. | Head or part of the trunk lies on the palm. | The trunk rests on the palm of the hand; the mouse leans to one side but may have difficulty regaining an upright position. | Upright position not possible. | 0-4 |
| Spontaneous Activity | General | Observation on open tabletop without interference | Mouse is alert and actively exploring. | Mouse appears alert but is calm and quiet. | Mouse starts and stops exploring slowly and repeatedly. Mouse is listless, moves slowly but does not explore. | Mouse is lethargic or stuporous and barely moves. | No spontaneous movements. | 0-4 |
| Body symmetry | Focal | Observation of undisturbed resting behavior and description of the virtual nose-tail line | Normal. Body: normal posture, trunk elevated from the bench, with forelimbs and hindlimbs leaning beneath the body. Tail: straight. | Slight asymmetry. Body: leans on one side with forelimbs and hindlimbs leaning beneath the body. Tail: slightly bent. | Moderate asymmetry. Body: leans on one side with forelimbs and hindlimbs stretched out. Tail: slightly bent. | Clear asymmetry. Body leans on one. Tail: clearly bent. | Complete asymmetry. | 0-4 |
| Gait | Focal | Observation on open tabletop without interference | Normal. Gait is flexible, symmetrical and fast. | Stiff, inflexible. Mouse walks hunchbacked and slow. | Limping with asymmetrical movements. | More severe limping, drifting, falling, with obvious gait deficits. | Does not walk spontaneously. Mouse takes only a few steps when stimulated. | 0-4 |
| Climbing | Focal | Mouse is placed in the center of a gripping surface at a 45° angle to the tabletop | Normal. Mouse climbs quickly. | Weak limbs, climbs slowly. | Stays on slope, does not slip or climb. | Slides down slope; difficulty in fall prevention. | Slides down slope, unsuccessful attempt at fall arrest. | 0-4 |
| Circling Behavior | Focal | Observation of the mouse walking undisturbed on the tabletop | No circling. Mouse turns equally to left or right. | Predominantly one-sided turns. | Circles sideways, though not constantly. | Circles constantly to one side. | No movements. | 0-4 |
| Forelimb symmetry | Focal | Mouse suspended by its tail. Movements and position of the forelimbs are observed | Normal. Both forelimbs are extended toward the bench and moving actively. | Slight asymmetry. Contralateral forelimb not fully extended. | Marked asymmetry. The contralateral forelimb is flexed toward the trunk. | Prominent asymmetry. Contralateral forelimb attached to trunk. | No body or limb movement, slight asymmetry. | 0-4 |
| Compulsory circling | Focal | Handstand position: Forelimbs on bench, hindlimbs suspended by the tail | No circling. Normal extension of both forelimbs. | Both forelimbs extended, but beginning to circle mostly to one side. | Rotates to one side only and can fall to one side. | Pivots sluggishly to one side and does not rotate in a full circle. Falls to one side. | No or rare movements. | 0-4 |
| Gripping of the forepaw | Focal | Mouse is held by the tail on the top of the cage so that the front paws touch the bars | Mouse grasps the grid firmly with the front paws and tries to place the hind paws on the grid as well by pulling the hind paws under the body. | Mouse accesses the grid but has less power. A light pull breaks the grip of the front paws. | Mouse cannot grip with the affected front paw. | Mouse cannot grab the grid. |  | 0-3 |
